# Community health workers' involvement in mother–child care during the 1st year after birth, in Kaya health district, Burkina Faso: A contribution analysis

**DOI:** 10.3389/fpubh.2022.938967

**Published:** 2023-01-11

**Authors:** Halima Tougri, Rachidatou Compaoré, Adja Mariam Ouédraogo, Blandine Bila, Marleen Temmerman, Séni Kouanda

**Affiliations:** ^1^Département Biomedical/Santé Publique, Institut de Recherche en Sciences de la Santé (IRSS), Ouagadougou, Burkina Faso; ^2^International Centre for Reproductive Health (ICRH) Ghent University, Ghent, Belgium; ^3^Aga Khan University, Nairobi, Kenya; ^4^Département d'Épidémiologie, Institut Africain de Santé Publique (IASP), Ouagadougou, Burkina Faso

**Keywords:** mother-child care, contribution analysis, community involvement, postpartum care, community health worker (CHW)

## Abstract

**Introduction:**

Maternal and infant morbidity and mortality remain high in sub-Saharan Africa. However, actions to strengthen postpartum care are still weak and mainly limited to health facilities (HFs). In Kaya health district, Burkina Faso, community health workers (CHWs) were involved in mother and child care during the 1st year postpartum through home visits, outreach sessions and accompanying mothers to health facilities. The aim of this study was to assess the contribution of CHWs to postpartum women's attendance at the health facilities.

**Methods:**

We conducted an effect assessment using Mayne and Lemire's contribution analysis framework. Qualitative and quantitative data were collected through project documents review and individual semi-structured interviews with key-informants.

**Results:**

All the participants interviewed acknowledged that the number of women, who came to postpartum care, had increased since the implementation of the project activities. Postpartum consultation rates within the 1st week postpartum increased from 29% in 2011 to 80% in 2015 and from 19 to 50% within 6 weeks. Others interventions such as Performance based financing, Save The Children nutritional project and the health services component of Missed Opportunities in Mother and Infant Health (MOMI) were the alternative explanations.

**Conclusions:**

CHWs involvement in women care contributed to improve their adherence to postpartum consultations in Kaya health district.

## Introduction

Maternal and infant morbidity and mortality remain high in the world and particularly in sub-Saharan Africa. In 2017, the number of women who died from pregnancy or delivery complications was estimated at 295,000 (1).

Almost all maternal deaths (94%) occur in developing countries and most could have been prevented. Sub-Saharan Africa and Southern Asia accounted for approximately 86% (254,000) of the estimated global maternal deaths in 2017. Sub-Saharan Africa alone accounted for roughly two-thirds (196,000) of maternal deaths, while Southern Asia accounted for nearly one-fifth (58,000) (1).

Although most maternal deaths occur between the third term of pregnancy and the 1st week after childbirth, women still face a high death risk beyond 6 weeks postpartum (2, 3).

In 2013, in sub-Saharan Africa, 17.6% of maternal deaths occurred in the intrapartum period and within the 24 h following delivery, 47.8% between 24 h and the 42nd day. Some women (13.1%) died from postpartum complication between the 43rd day and a year after delivery (4).

Despite a clear pattern of mortality and morbidity during the postpartum period, actions to improve postpartum care remain poor and are mostly implemented at the health facility level, sometimes not including the communities (3).

However, since the Alma Ata conference, communities have been largely involved in the health systems in most developing countries through community health workers (CHWs).

Studies on community involvement in the improvement of maternal and child health in general, and specifically in the postpartum period, have already been conducted in several countries through different approaches (2, 4–7). The evaluation of these programs has shown that CHWs can contribute to community health development by acting as a bridge between the community and health services.

However, most of these evaluations have focused on the effectiveness, feasibility (8, 9) or impact of these programs (10). Few impact evaluation studies have examined the context and mechanisms that led to the observed results (11, 12). In addition, the majority of these evaluations used counterfactual elements or control vs. intervention groups to attribute an observed result to a given program (13). However, in complex interventions, it is often difficult to fully attribute observed effects to the intervention (14–17). It would therefore be interesting to determine the contribution of each component of an intervention or a programme to the achievement of the obtained results, in order to optimize and improve strategies for public health interventions (11, 12, 14).

Missed Opportunities in Mother and Infant Health (MOMI) project was implemented in four countries of sub-Saharan Africa, including Burkina Faso in 2011 for 5 years; the project aimed at improving maternal and child health with a focus on the year following birth through two components, community and health services.

Our study aims to analyse the effect of the intervention of community health worker as component of the MOMI project in the Kaya health district in Burkina Faso, by using a mechanistic approach rather than a counterfactual one.

The specific aims of this study are to analyse the contribution of CHW involvement in the achievement of the effect, in particular the attendance of health facility in postpartum women; to identify the factors that have contributed to or limited women's access to postpartum health services; and to identify elements of the intervention that contributed to health facility attendance by women during one the year after birth.

## Description of the MOMI project

The Missed Opportunities in Maternal and Infant Health (MOMI) project is funded by the European Union and implemented in four African countries: Kenya, Malawi, Mozambique and Burkina Faso. In Burkina Faso, the project was implemented in 12 health facilities in Kaya health district, including eight rural and four urban HF. These 12 health facilities were selected based on the low rate of postpartum consultation and their location in the Kaya health demographic site (Kaya HDSS) (18). The objective of the MOMI project at the community level was to improve maternal and child health through women's adherence to postpartum consultations. Through the involvement of the CHWs, the project aimed at increasing the rate of postpartum consultations in these 12 health facilities. These CHWs were the same individuals selected by the community and were already working in collaboration with health workers. They were involved in health promotion and their routine activities were only focused on pregnant women (accompany some women for delivery, home visit...) but were not interested in postpartum women.

MOMI project introduced activities for postpartum women and their child (home visit, outreach sessions, reference of mother/child to HF and accompaniment for postpartum consultation). However, because of the context, only female CHWs were involved in MOMI project activities. These CHWs were trained during 3 days and retrained annually until the end of the project. Using picture boxes, the training focused on prenatal consultations, assisted deliveries, family planning, post-natal consultations, recognition of danger signs in mothers and children, the tasks of CHWs in the MOMI project, and data collection tools for CHWs.

The MOMI project included three intervention components selected through a participatory process. One component was implemented at the community level; specifically, the support of the mother-infant by the CHWs in the 1st year following delivery. The other two interventions were implemented at the health facility level—specifically, improved immediate postpartum care and integration of maternal and infant health services (19).

At the community level, each CHW had to carry out three home visits to any mother and infant in her coverage area during the 1st year following delivery. The first one was before the 10th day from the delivery, the second visit was between the 6th and the 8th weeks and the last visit was between the 9th and the 12th months.

These home visits corresponded to the different periods of postpartum consultation or immunization at the health facility level. In our context, according to the ministry of health schedule, the first postpartum visit occurs between the 6th and 8th day after delivery and the second between the 6th and 8th week. Vaccination against measles takes place between the 9th and the 12th month.

During home visits, the CHW had to take care of both mother and child, look for signs of danger and refer if necessary. They must also refer home delivery women to the HF. Depending on the visit time period, using a picture box, the CHW also carried out outreach sessions on the signs of danger, family planning, and the importance of postpartum consultations, etc. Outreach sessions were carried out in households, at HFs, during wedding or baptism ceremonies, or during any other activity involving a group of women.

As much as possible, the CHW was to accompany the woman to the health facility for postpartum consultations.

The project was initiated in 2011 with a situational analysis. After the stakeholders training, the implementation of activities at the community and health facility level began in September 2013 and ended in December 2015.

CHWs were supervised 1 month after the training and then every 3 months until the end of the project. This supervision was led by the project team in collaboration with the Kaya health district management team.

The project opted for a non-financial motivation, CHWs were not provided any incentives for carrying out the activities. They received bicycles at the project beginning and during each supervised session, meeting and training, they were compensated with a daily subsistence allowance and transportation fees. They received certificates of participation at the end of the project.

## Materials and methods

### Study setting

The MOMI project was implemented in the Kaya health district, which is located in the Center Nord health region of Burkina Faso. The health district of Kaya covers 342 villages and seven urban areas. This district is centered on a regional hospital center (CHR) with 60 health facilities, including 54 health and social promotion centers (CSPSs) and a central private medical center with surgical satellite facilities (CMA). Kaya Health District is a pilot site for several reproductive health interventions, but reproductive health indicators remain low (18).

At the community level, there are two CHWs per village, usually a man and a woman responsible for carrying out health promotion activities and malaria case management, diarrhea, etc.

### Study design

We carried out multiple case study design using a mixed method approach (qualitative and quantitative). The cases were the sites where the intervention was implemented. The cases chosen are contrasted according to location (urban vs. rural) and performance (low vs. high). The indicators used for the performance classification were: ≪ Proportion of mother/infant pair who received postpartum care at the health facility ≫, ≪ Proportion of mother/infant pair who received CHWs home visit ≫. We mobilized longitudinal quantitative data, cross-sectional qualitative data and document review. The qualitative study was conducted from June to September 2015.

### Sampling approach and sample size

The sample for the quantitative study included all 12 study sites that were selected for intervention implementation, based on maternal health indicators in the Kaya health district. All the 72 CHWs involved in the MOMI project were also included. The present study focused on the community level component of the MOMI project.

For qualitative study, we use purposeful sampling to select four cases sites taking into account geographical variation and implementation degree. So, two sites with low implementation effectiveness and two with high were selected. As many of providers as possible were interviewed with the objective of interviewing at least two per health facility. In addition, a minimum of two community health workers assigned to each facility and a minimum of three women postpartum, one who had delivered in the facility, one who had delivered at home and one who was attending for infant vaccination were also interviewed. The numbers of interviewees determined aimed to sample with maximum diversity whilst minimizing the number to what was feasible in terms of research capacity. In addition to the case study interviews, key members of the MOMI teams and other stakeholders who had been involved throughout the period of implementation were interviewed to track the process of implementation and identify barriers and facilitators along the way. A more detailed overview of the data collection is available elsewhere (20).

As for saturation, we listened to some of interviews again to ensure that the information sought according to our objectives had been captured. In addition, we re-read notes taken during the interviews to ensure the completeness of the information.

### Data collection

We collected non-concomitant quantitative and qualitative data.

#### Quantitative data

Quantitative data on CHW's activities and women postpartum attendance were collected quarterly by the research team through the tools put in place within the project framework: the ideogram, reference card, CHW diaries and postpartum care records. Data on women HF attendance concerned the period before and after the intervention in the 12 health facilities were collected monthly, 12 months before the implementation of the intervention (Sep 2012 to Aug 2013) and 27 months throughout the project (Sep 2013 to Dec 2015). CHWs' activities data were collected during the implementation period through CHW's diaries, referral cards, and report. Data were captured by the CHWs using diaries and then compiled by them through ideogram a kind of monthly record. These monthly report are compiled by research team every 3 months into Excel file.

The variables of interest were the proportion of home visits from a CHW, postpartum women accompanied to the HF for post-natal consultation, mother/child reference to health facility for danger sign, postpartum consultations in health facilities and the number of sensitizations.

#### Qualitative data

Qualitative data were collected by the researchers with master degree in sociology, through semi-structured interviews and document reviews. The interviews were conducted in French and Mooré (local language) at the HF level and in the policymaker's office, using an interview topic guide. They were all audio-recorded with participants' permission, lasting between 30 and 75 min.

The data sources for document review included supervision reports, policy advisory board (PAB) meeting minutes, stakeholder and community leader meeting minutes, the critical review of the policy report (Work package or WP2) (21), the situational analysis report (WP3) (22), the selection of interventions report (WP4) (19), and the event log.

### Data analysis

Quantitative and qualitative data were analyzed separately. The themes that emerged from the qualitative analysis were compared with the quantitative data in the same facilities to determine the plausibility of the program theory given the data.

### Theoretical framework

For our evaluation, we used the six steps of the contribution analysis framework proposed by Mayne, combined with Lemire's framework, the Relevant Explanation Finder (REF) (23).

Based on the observation that the rates of postpartum consultations had increased since the introduction of the MOMI project interventions in the Kaya health district, we developed cause-effect questions, which we would answer to throughout the analysis (Steps 1–3):

- Did the involvement of the CHW in the care of women and their children contribute to increasing the use of postpartum health services by women?
- Which factors contributed to or limited the use of health services by women during the postpartum period?
- What aspects of the intervention have contributed?

**Figure 1:**
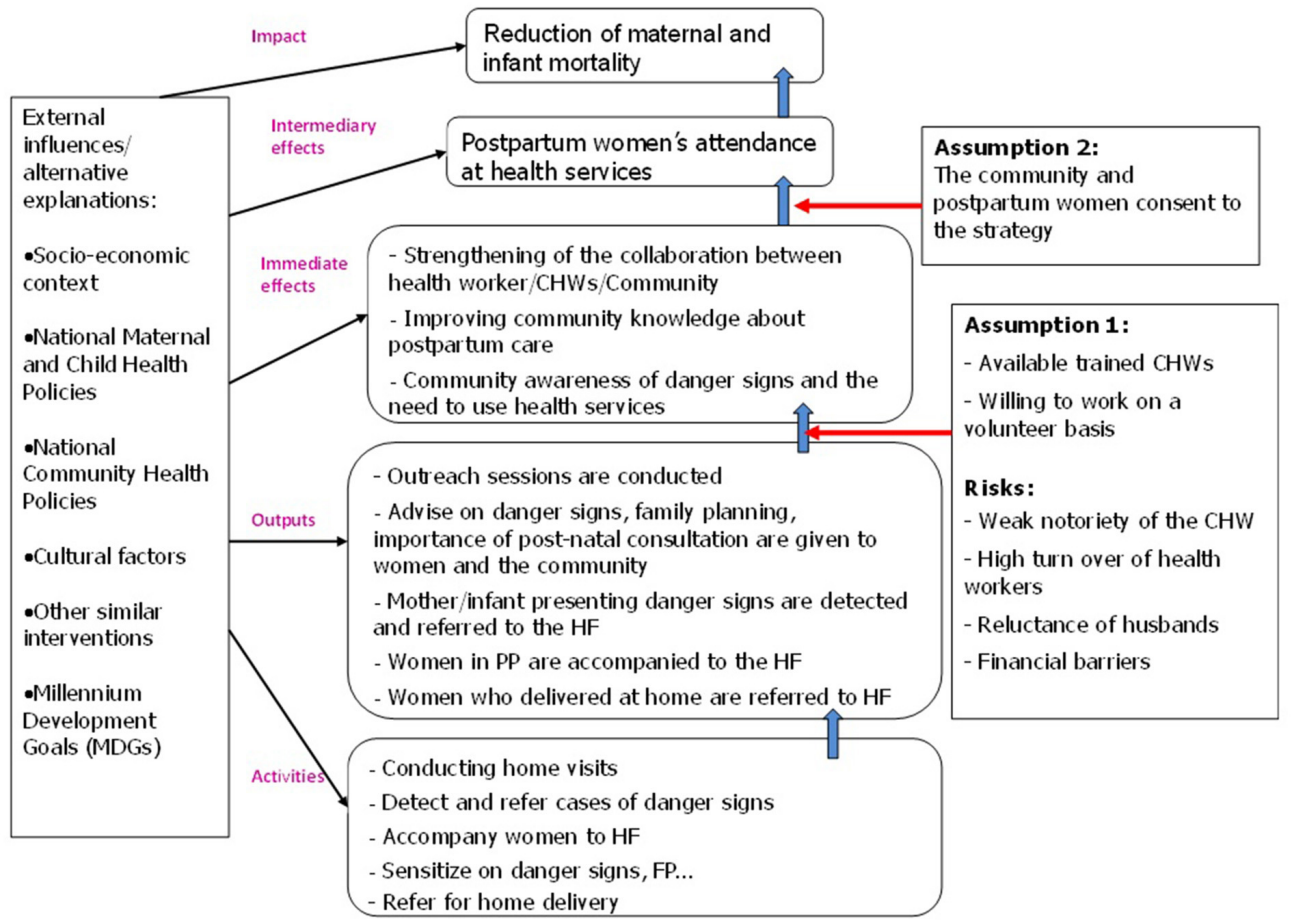
Logic model of the MOMI project at the community level. Adapted from Mayne's 2012 model, quoted by Stocks-Rankin.

To answer these questions, we retrospectively developed the programme theory at the community level (Figure 1) and then tested the assumptions contained therein.

Then we used Lemire's framework, REF (steps 4, 5), to examine the mechanisms of change and to understand the influencing factors and alternative explanations. REF components are presented in Table 1.

**Table 1:** Relevant explanation finder (REF).

**Assumptions**	**Mechanism**	**Influencing factors/alternative explanations**	**Type of rival**	**Level**	**Degree of influence**				
					**Certainty**	**Robustness**	**Range**	**Prevalence**	**Evidence**
Trained CHWs are available and agree to conduct home visits, outreach sessions and accompany women to HFs without being paid	Promotion activities led by CHWs boost their trust in the role they are playing for the improvement of PPCs	The participatory approach used by the project to select interventions and CHWs	Primary rival	Structural	High	High	High	High	High
	CHW support systems such as trainings, supervision and non-financial incentives build their capacities and encourage them to carry on their activities.	The adherence of CHWs and health workers to the project strategy	Primary rival	Individual	High	High	High	High	High
	The CHW is a community member; therefore, she can easily conduct home visits, and advise, she can be more trustworthy, and she can serve as link between the community and health facilities	Collaboration between CHWs and health workers	Primary rival	Interpersonal	High	High	High	High	High
		Motivation system adopted by the project	Commingled rival	Individual	Medium	High	Medium	Medium	High
		The educational level and social status of some CHWs	Direct rival	Individual	Low	Low	Low	Low	High
	The interventions are carried out in order to motivate community leaders to get more involved.	Cooperation of the Policy advisory board (PAB)	Implementation rival	Institutional	Medium	Medium	Medium	Medium	High
		Monitoring/assessment of the activities	Implementation rival	Institutional	High	High	High	High	High
		Mother and child health national policy	Implementation rival	Institutional	Medium	Low	Medium	Low	High
Women who delivered in the community are receptive to the advice given by CHWs; they understand and apply recommendations.	The CHW represents a link between the community and health service, which helps remove barriers such as fear and suspicion of the community toward health workers.	Postpartum women's attitude, enthusiasm and interest in postpartum care	Primary rival	Individual	High	High	High	High	High
		Trust between the CHWs and women	Primary rival	Individual	High	High	High	High	High
	Women's adherence depends on their husbands or their mothers-in-law.	Weak decision-making power of women mainly in rural areas	Primary rival	Institutional	Medium	Medium	Medium	Medium	Medium
	Behavioral change is adopted when postpartum care is promoted within the community.	The low purchasing power of some women represents a financial barrier	Implementation rival	Individual	Medium	Medium	Low	Low	Medium
	Women who were supported by CHWs share their experience with other women.	Peer-to-peer education	Implementation rival	Individual	High	Medium	Medium	Medium	High
	Men are more receptive to men's or leaders' messages	Involvement of the community through community leaders and male CHWs	Implementation rival	Institutional	High	Medium	Medium	Medium	High
	The attendance at health facilities can be motivated by other interventions.	HF component with inclusion of care for mother and child.	Implementation rival	Institutional	High	High	Medium	Medium	High
		PBF with more involvement of health workers	Implementation rival	Institutional	Medium	Medium	Medium	Medium	High
		Food distribution by the project VIM to women who attend HF appointments	Implementation rival	Institutional	High	Medium	Medium	Medium	Medium

Finally, we wrote the contribution story (Step 6), which is an iterative process of identifying the elements of the intervention that contributed to increase postpartum women's use of health services.

**Figure 2:**
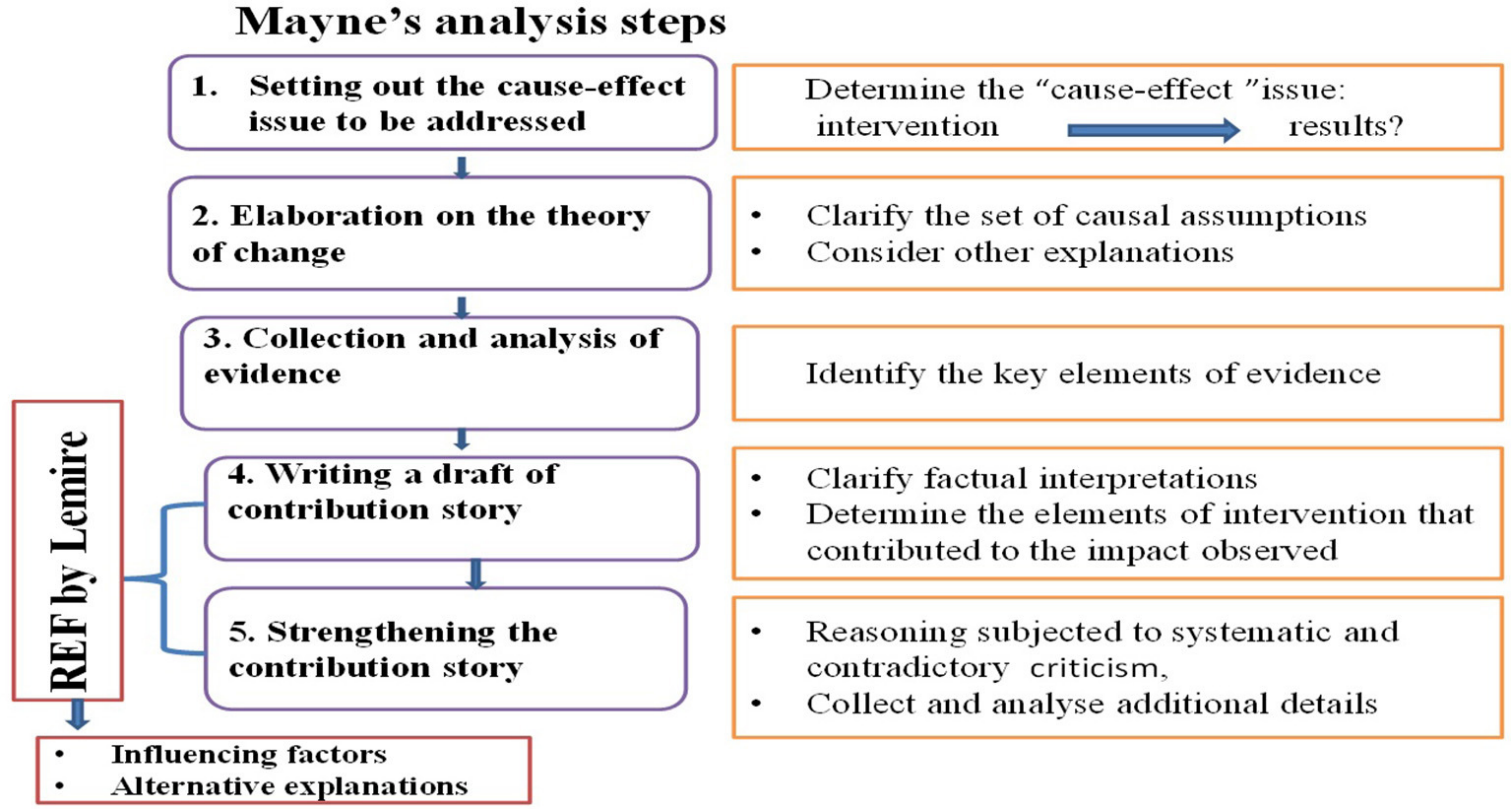
Conceptual framework: contribution analysis.

To reinforce the plausibility of the results of the contribution evaluation, our analysis focused on the two main assumptions used in the REF (Table 1). The conceptual framework of contribution analysis is represented in Figure 2.

### Quantitative data

Quantitative data were entered using Epi data and analyzed with Excel 2007 and Stata 13 software. We realize descriptive statistical analyses. Women HF attendance was represented by monthly proportion of pair mother-child received at the HF for postpartum visit. The monthly proportion of mother-child who received CHW visit or accompaniment designed CHWs activities.

### Qualitative data

All interviews were numerically recorded, then transcribed in verbatim by qualified transcribers. Each transcript was translated into French for those who were in local language, and coded with Nvivo 11 software by the researcher team. We performed a content analysis based on the themes from the conceptual framework (contribution analyzis).

Findings were discussed and triangulated between research and evaluation team to identify influencing factors, alternative explanations and contribution elements. The contribution story was then written by a member of the research team. This narrative was then discussed iteratively among the research team, evaluators, and resource persons from health ministry to identify the real contributions of CHWs to women's use of health services.

To avoid conflict, researcher who conducted evaluation are different from those who conduct implementation.

The quantitative method measured the degree of implementation, and the qualitative method enabled us to assess the context and collect additional details on the degree of implementation through the document review and the interviews. Thus, by triangulation, the factors affecting the implementation, the alternative explanations and the elements of contribution were identified.

## Results

### Characteristics of the studied population

Of the 72 CHWs trained, 65 completed the activities to the end of the project. The majority of them lived in rural areas (84.6%) with a low level of education (66.2%) and used to accompany women to health facilities for childbirth prior to the installation of the MOMI project (61.5%). The average age was 47 (±10) years. Length of service ranged from 3 to 32 years with an average of 11 (±8) years.

A total of 49 participants were interviewed during the case study, including 13 CHWs, 13 postpartum women, 16 health workers, 04 policymakers, and 03 project team members in Burkina Faso. The health workers included four head nurses, three maternity officers, four Expanded Program on Immunization (EPI) officers, and five other staff. Among the rural participants were six postpartum women, five CHWs, and six health workers.

### Contribution analysis (CA)

#### Applying Steps 1–3 of contribution analysis

The application of Steps 1 to 3 helped to define the causal issue, develop the theory of change, and gather evidence to support the hypotheses made. The evidence collected at this step is the activities carried out by the CHWs and the use of postpartum services by women.

#### Intervention activities carried out by the CHW

**Figure 3:**
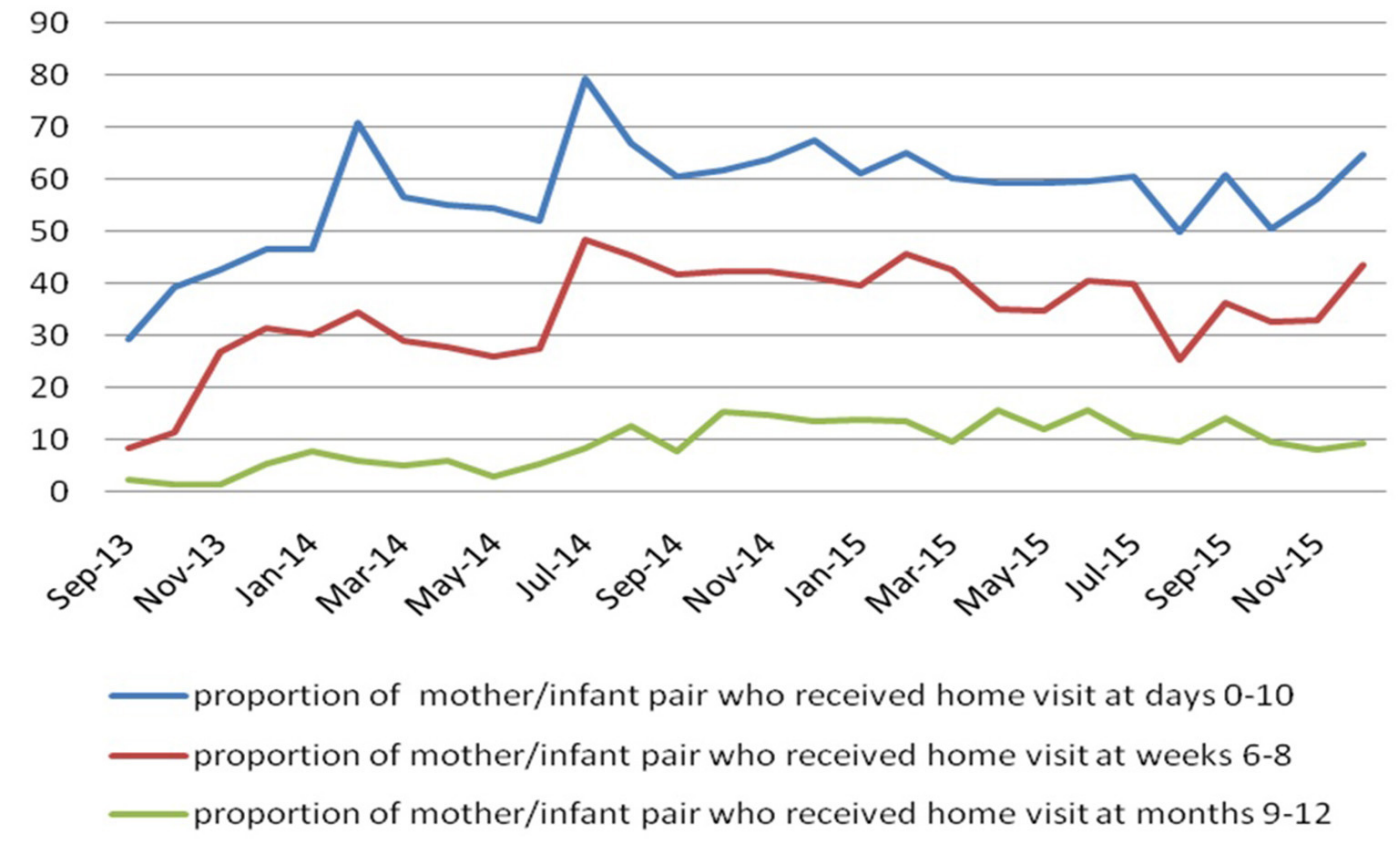
Home visit evolution per month from September 2013 to December 2015.

The proportion of women giving birth who were visited by CHWs or accompanied to a health facility varied according to time period and setting (>50% in rural areas). Thus, 56.8% (6,633/11,675) of women received the first visit, 34.3% (3,999/11,675) the second visit and 9% (1,067/11,675) the third visit. Figure 3 shows the evolution of home visits by month from September 2013 to December 2015. During the same period, respectively, 57.3% (3,803/6,633), 51.4% (2,056/3,999) and 32.5% (342/1,067) of women were accompanied to health facilities. A total of 5,681 awareness-raising activities were carried out.

#### Women's attendance at health services

**Figure 4:**
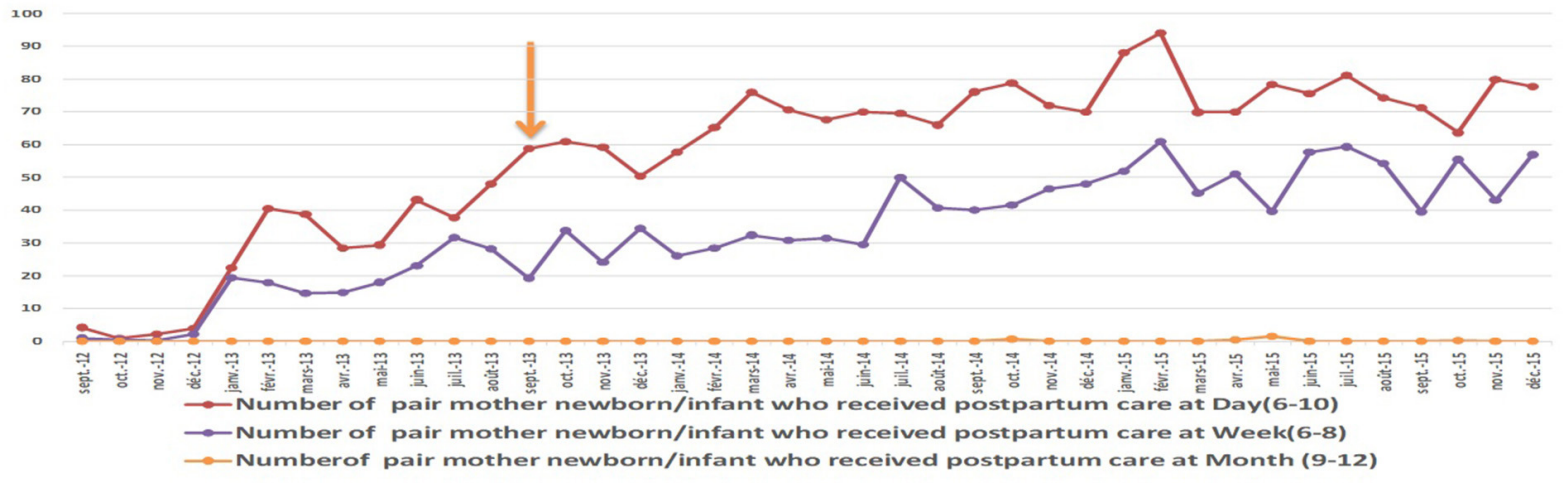
Proportion of pair mother /infant who received postpartum care before and after the intervention.

The 6th day's consultation rates increased from under 40% at the beginning of the intervention to 80% after, and the 6th week's consultation (42nd day) rates increased from under 20% to over 50% for all the health facilities. All of the participants interviewed in this case study acknowledged that the number of women who came to postpartum care, including those who gave birth at home, had increased considerably since the implementation of the project activities. The observations during supervision and the data collected during monitoring confirmed this assertion. In addition, health workers who were interviewed reported that the number of women who received postpartum consultations was higher on the 6th day than in the 42nd day (Figure 4).

#### Applying the relevant explanation finder (Steps 4, 5)

These steps were used to understand the influencing factors and alternative explanations.

#### Influencing factors

Among the facilitating factors, we noted the participatory approach adopted by the project for the selection of CHWs and their activities, CHWs commitment, the involvement of community leaders, the health workers collaboration, the non-financial motivation system, the influence of the other women and regular supervision of the activities.

- *CHWs' commitment and motivation*

The majority of CHWs (90.3%) conducted the activities until the end of the project. Their main motivation was the moral duty toward the community that had chosen them because they felt valued by this community. They had the conviction that their work had a positive impact on maternal and child health because they thought they were influencing women in the use of health services.

During the interviews, some of them expressed themselves as follows:

*I agreed to do this work because when the members of your community meet and choose you to entrust you with a responsibility, you have the duty to do it otherwise it will seem like you are not concerned about people's health. (CHW from a rural HF)*

*It's for our well-being because health is priceless. And also, it is our village, and we have the duty to contribute to its development. If we are concerned about the development of our village, we must wholeheartedly support the well-being of the population. This is the reason for our involvement in contributing to the improvement of health. (CHW2, Rural HF)*

However, 9.7% of CHWs left the project because of the non-financial incentive. During supervision and interviews, a CHW from the urban area who left the project expressed herself:

*I warned you from the beginning that your volunteering story was not going to go far. Anyway, I am not visiting women anymore because we earn nothing in your project. I manage with my small business to feed my children and you want me to stop this and go visiting women with nothing in exchange? (CHW, urban HF)*

- *CHWs' bridging role*

The baseline study showed that women's acceptance of the CHW would depend on her reputation within the community and her collaboration with health workers. *Via* this relationship, the CHWs conducted home visits and outreach sessions. Data from the various interviews confirmed this assumption.

*Our CHW helps us a lot. Even at night, when a woman is in labor, she accompanies her to the HF without any hesitation. Sometimes she gets on her bike to follow (the woman) to the HF. (Postpartum woman, rural HF)*

The messages they conveyed were welcomed by women. The women interviewed described them as a benchmark for maternal health in the village and advocates for women in interactions with their husbands and health workers. Their presence broke down barriers such as fear and mistrust that some women might feel in the presence of a health worker.

The following statements collected during the interviews confirmed these facts:

*There are women who want to go to the HF, but they are afraid of health workers. But they say that if the CHW working with the health workers accompanies them, they will no longer be afraid and will express themselves freely because the CHW is there. (ICP, urban HF)*

- *Involvement of community leaders*

The involvement of community leaders and male CHWs facilitated the work of the CHWs in the field. Several CHWs interviewed reported that the community members consented to participate in their activities since the community leaders themselves were involved in the project. Thus, they had access to households for outreach sessions, and women were receptive.

“*They [women] simply believed that it is for their benefit because an activity that village leaders and authorities are involved in can only be beneficial to the community. So, they consent as well.” (CHW6, urban HF)*

However, other factors have limited women's access to these services, such as geographical and financial accessibility (especially during the rainy season), and the weak decision-making power of women, especially in rural areas. Women, health workers and the CHWs interviewed confirmed these obstacles.

*Here in the city, there is no problem because women are autonomous. But in the villages, men decide where, when and how their wives can go. They decide whether their wives must go to the HF or not. Sometimes women or children can fall sick for more than a week, but they are waiting because their husbands did not give permission to go to consultation. (Health worker, urban HF)*

During the monitored supervision sessions, the CHWs reported that some women did not come to postpartum consultations because of the cost of consumables.

*You know, madam, I will tell you the truth. Our women do not come on the 42nd day because your health workers asked them to pay for gloves, speculum, etc. So, if you do not have money and your husband also refuses to give you some, you have to stay home. (Rural CHW)*

#### Alternative explanations

The health services component of the MOMI project, the performance-based funding (PBF) and the Save the Children NGO's project called “Victoire sur la Malnutrition” (VIM) implemented in 2014, were alternative explanations.

Indeed, the health services component of MOMI advocated the integration of maternal and infant health care so that some health workers would include postpartum visits with mothers and their infants during immunization visits or healthy infant consultations. For example, in some health facilities, appointments for postpartum visits on the 6th day were scheduled at the same time as the child's BCG vaccination, and the 6th-week appointments were scheduled at the same time as the child's PENTA1 vaccination or healthy infant consultation appointment. In addition, within all the health facilities, the health worker keeps the mother's health record after delivery. The records were delivered only on the sixth postpartum day. An urban health worker said the following during supervision:

*Since we are keeping the health records, women come back, if only for their health records, and we seize this opportunity to treat them. But sometimes we are obliged to hand over the notebook before the consultation when the woman is not from the same location. (Supervision Report)*

The performance-based financing (PBF) was funding some maternal health indicators, such as postpartum consultation rates. This led some health workers to become more involved in postpartum services. Their collaboration with the CHWs in the search for women who were absent at the various appointments was intensified.

The VIM project distributed food to pregnant and breastfeeding mothers in rural areas. This distribution was based on the mothers' adherence to prenatal consultations and the immunization schedule for children. Another influence of the VIM project may lie in the fact that some CHWs who worked in the MOMI project were also working on the VIM project. These CHWs took advantage of the food distribution incentive to sensitize women. These facts were corroborated by supervisory data and interviews.

*We also take advantage of the distribution of VIM to raise awareness on FP (family planning), the importance of meeting (health consultations) appointments, and signs of danger (for mother and child's health). We generally finish our sensitization before we start to share food; otherwise, women may leave. (Supervision Report)*

#### Assembling the performance story of contribution (Step 6)

This is an iterative process of identifying elements of the intervention that contributed to increasing the attendance of health services by postpartum women. Among the causal links established in the theory of change, we believe that the participatory approach adopted by the MOMI project for the identification of the activities and the selection of the CHWs enabled the appropriation of the intervention by the different actors. The involvement of the community through male CHWs and community leaders helped boost female CHWs in their actions in the sense that they felt valued and considered. Regular training and supervision boosted the CHWs' self-esteem and confidence.

This perception of the importance of their role motivated them to carry out the project activities among women. These activities enabled women to become aware of the dangers that could arise after delivery and the importance of postpartum care at the health center, which explained their agreement to attending postpartum health services.

However, of all the activities carried out, we believe that home visits and outreach sessions, as well as a good collaboration between health workers and CHWs, contributed more to the attendance of health services by women.

On the other hand, the accompaniment of women to the HFs seemed to have a smaller contribution because this activity was not carried out by all the CHWs because of the lack of adapted means of transport: while the CHWs were riding a bicycle or were walking, the postpartum woman would ride a motorcycle or a bicycle.

## Discussion

Relevant Explanation Finder element analysis showed that there was a link between the activities of the project and the use of health services by women during the postpartum period. The analysis also showed that some factors contributed to, or limited women's access to health services; however, other factors might also explain the increase in the use of postpartum services.

### Activities and influencing factors

During the project implementation, most of CHWs provided home visits, accompanied women in health facilities and carried out outreach sessions. These activities varied according to the period and the health facility. In general, the health facilities located in rural areas had a home visit rate of more than 50%, while in urban areas are <50%. The participative approach adopted by MOMI project to select the intervention activities and CHWs and the commitment of the CHW can explain these results. Community involvement in the selection of interventions was significant in facilitating the work of community health workers within villages (24–26). In fact, community recognition was the main source of motivation of several CHWs to conduct the project activities. This has also been observed in several studies conducted in Nepal, Gambia and Ethiopia (25, 27, 28). CHWs were selected by their community, and they represented the resource persons of the community in these locations. Therefore, they were motivated to help their community in matters of health. We found, as in the other MOMI project countries (Kenya, Malawi, Mozambique), that the trust between CHWs and their community motivated them to provide an effective bridging function (12). The mutual trust between the CHW and her community, as well as the collaboration between the CHW and the formal health workers, made a bridge between the community and health services. The CHW was perceived as a real asset by the community and health services. The messages they conveyed were welcomed by women. However, CHWs were more respected and paid more attention to by the population in rural than the urban areas. This may explain the high rate of visits observed in rural areas. Other factors may also explain the high rate of visits, particularly the seniority of the CHWs, their advanced age and the dynamics of the voluntary CHWs in the health facility. In addition, elder CHWs had no young children, were supported by their loved ones and were freer to provide home visits than young CHWs. Sensitization through social networks with an appropriate timetable as implemented by the female CHW proved positive for behavioral change and raised attention to PPC (29, 30). Monitoring and supervision were revealed to be an important factor of success for the interventions (12).

Women in rural areas had a higher rate of home visits than women in urban areas. This situation in urban areas could be explained by the difficulties encountered by the CHWs in conducting home visits, particularly difficulties relating to their social status. Therefore, the scaling up of such intervention must take this reality into account and consider only the rural environment in order to ensure the success of the intervention. Monica et al. found in a similar study in Uganda that the poor education level of community health workers negatively affected their activity in urban areas (31).

### Alternatives explanations

On the other hand, the alternatives explanations to the health services component of the MOMI project, the performance-based financing (PBF) and the Save the Children NGO's project called “*Victoire sur la Malnutrition*” (VIM). Belemsaga et al. in their study had shown that key contextual factors of the successful upgrade of postpartum care interventions in Burkina Faso were the retention of the health book at the health facility until the day 6–10 visit (10). The fact that the PBF buys certain postpartum indicators (6th and 42nd day), health workers have stepped up their monitoring of postpartum women and strengthened their collaboration with CHWs in the search for those lost to follow-up. This has contributed to women's use of health services.

### Contribution story

PBF and VIIM were mainly implemented at the health facility level. Moreover, these projects were established well after MOMI project and do not have community activities, although they are interested in postpartum women. The postpartum indicators collected in the HFs before and after the implementation of MOMI showed an increase in the use of postpartum services since the implementation of the project. This trend continued with the implementation of the other projects. That suggest that MOMI contributed to the observed effects, and the contribution elements could be attributed to CHWs activities, including home visits, outreach sessions, etc.

### Sustainability

One of the difficulties encountered in implementing the interventions at the community level was the financial incentive of the CHWs. It was very difficult to find the kind of motivation that would suit everyone. While some people were advocating fee-for-service, others opted for non-financial motivation such as being equipping with a bicycle (32). To ensure the sustainability of the intervention, the project team had opted for a system of non-financial motivation of CHWs. But this type of non-financial incentive had a mixed impact on the project implementation. In fact, it encouraged the majority of CHWs to carry out the activities, but it also resulted in the resignation of 9.7% of them. Studies conducted in Nepal and South Africa reported similar results (27, 33). In Tanzania and South Africa have studies had shown that while money was an incentive, non-financial learning incentives were favored (25, 27, 28). However, in 2016, the ministry of health decided to motivate the CHW with 60 USD (30 000 XOF per month).

The involvement of CHWs in the management of women during the postpartum period contributed to increasing the attendance of these women in health facilities. Several factors helped achieve these results, including the non-financial motivation system. However, although the system adopted by the MOMI project did not have a negative impact on the majority of CHWs, it should be noted that it is difficult to continuously work without being paid in a context of poverty. It is time to find a permanent mechanism to motivate CHWs or find other adapted interventions to ensure the sustainability of interventions involving the community. This could include the pooling of resources and integration of activities of all NGOs working in the field with the same CHWs.

## Limitations

The limitations of our study were mainly related to the evaluation approach used, that is, the contribution analysis. How a contribution analysis-based approach can account for the “cause-effect” relationship remains controversial. However, the methodology used enabled us to collect high-quality data and reliable information that could raise questions for further research.

The benefit of the CA (contribution analysis) is that it focuses on examining contextual factors and other explanations that affect the success and outcomes of the programme.

## Data availability statement

The original contributions presented in the study are included in the article/supplementary material, further inquiries can be directed to the corresponding author.

## Ethics statement

The study received the approval of Ethics Committee for Research in Health of Burkina Faso under the deliberation number: 2015-5-074. This committee belongs to the health ministry. We obtained written informed consent and guaranteed the confidentiality of all participants prior to the interviews.

## Author contributions

MT and SK designed the study. HT wrote the first paper. BB and RC analyzed the qualitative data. HT and AO the quantitative data. All authors review and approved the final version of this article.
